# Modulating the transcriptomic profile of multidrug-resistant *Klebsiella pneumoniae* biofilm formation by antibiotics in combination with zinc sulfate

**DOI:** 10.1186/s12941-023-00634-7

**Published:** 2023-09-12

**Authors:** Rania I. Shebl, Walid F. Elkhatib, Mona Shaban E. M. Badawy

**Affiliations:** 1 Department of Microbiology and Immunology, Faculty of Pharmacy, Ahram Canadian University, 6th October City, 4th Industrial Zone, Giza 12451, Egypt; 2 Microbiology and Immunology Department, Faculty of Pharmacy, Ain Shams University, African Union Organization St., Abbassia, Cairo 11566, Egypt; 3 Department of Microbiology and Immunology, Faculty of Pharmacy, Galala University, New Galala City, Suez, Egypt; 4 Department of Microbiology and Immunology, Faculty of Pharmacy (Girls), El-Azhar University, Cairo, Egypt

**Keywords:** *Klebsiella pneumoniae*, Multidrug-resistant, Antibiotics, Biofilm, Zinc sulfate, Combination

## Abstract

**Background:**

*Klebsiella pneumoniae* is a significant healthcare-associated pathogen. We investigated the antimicrobial interaction pattern between zinc sulfate and antibiotics against *K. pneumoniae* biofilm on the phenotypic and genotypic levels.

**Methods:**

Determining the minimum biofilm inhibitory concentrations and the transcriptomic profile of *K. pneumoniae* biofilm formation genes post-treatment were carried out to evaluate the effect on the phenotypic and genotypic levels, respectively.

**Results:**

Zinc enhanced the antibiofilm potentials of cephalosporins, aminoglycosides, and ertapenem, whereas it antagonizes the effectiveness of fluoroquinolones and meropenem on the phenotypic level. On the molecular level, zinc enhanced the anti-biofilm efficacies of cephalosporins (cefotaxime, ceftriaxone, ceftazidime, cefpirome, and cefepime) via down-regulating the expression of biofilm-related genes by 18-, 38-, 5-, 77- and 2-folds, respectively. Zinc in combination with aminoglycosides (kanamycin, gentamicin, and amikacin) reduced the expression of biofilm-related genes by 40-, 2602- and 20-folds, respectively, and by 2-folds in combination with ertapenem. However, a reduction in the down-regulatory potentials of fluoroquinolones was recorded following combination with zinc by 2-, 2-, 15- and 14-folds, respectively, and an up-regulation in the expression levels of the tested genes by 2-folds in the case of zinc/meropenem combination.

**Conclusions:**

Results revealed variable interaction patterns between different antibiotics in combination with zinc. Current findings also shed light on the antibiofilm potentials of zinc/antibiotics combinations especially when combining zinc with fluoroquinolones or meropenem to avoid their antagonistic effects.

## Background

Among various biofilm-forming pathogens, *Klebsiella pneumoniae* has been categorized as a crucial Gram-negative, encapsulated, non-motile Enterobacteriaceae. *K. pneumoniae* typically colonizes multiple sites from where it can reach the systemic circulation resulting in severe infections either in the surgical sites or in the urinary tract. It plays a key role in ventilator-associated pneumonia as well as in hospital-acquired pneumonia. *K. pneumoniae* infections are also of special concern, especially in the case of immunocompromised neuropathic and diabetic patients [1].

Many factors contribute to the virulence of *K. pneumoniae* such as the presence of capsular polysaccharides, outer lipopolysaccharide, and outer membrane proteins in addition to type-3 fimbriae, where the microorganism relies on these factors to survive and to escape from the immune mechanisms during infection and development of bacterial biofilm [2]. The biofilm formation potential of *K. pneumoniae* is of critical importance as it enhances its resistance to several antibiotics. Moreover, the rapid spread of the biofilm-forming multidrug-resistant (MDR) *K. pneumoniae* was demonstrated to be associated with an increased incidence of nosocomial infections leading to huge records of morbidity and mortality [3]. The recorded elevated incidence of MDR *K. pneumoniae* is also associated with the absence of a rationale use of antibiotics [4].

Biofilms are composed of polymeric matrix enclosing bacterial communities which are characterized by their high resistance to antibiotics [5]. This elevated resistance to antibiotics is attributed to the diminished rate of bacterial reproduction within the bacterial biofilms in addition to the reduced ability of the antibiotics to penetrate these biofilms. Therefore, the eradication of biofilm-associated infections is very challenging and could result in serious complications for patients in clinical settings [6].

It is worth noting that *K. pneumoniae* virulence genes were found to be also engaged in biofilm formation. Examples of these genes are *cps* and *mrk* genes which are involved in capsule formation and production of type 3 fimbriae, respectively. In addition to *wbbm* and *wzm* cluster genes which contribute to the synthesis of O-antigen that is present in the bacterial lipopolysaccharide and consequently in the production of *K. pneumoniae* biofilms. It was also demonstrated both *luxS* gene which is responsible for the regulation of type-2 quorum sensing and pgaABCD operon which synthesize and translocate poly-β-1,6-n-acetyl-d-glucosamine adhesion are contributors to biofilm production via stimulating the intracellular communication and enhancing the cellular binding to abiotic surfaces, respectively [7].

Owing to the urgent need for alternative antimicrobial options, the application of combined therapy has attracted the attention of many scientists and clinicians as an effective strategy to overcome infections associated with biofilms. This approach might be addressed via the combination between conventional antibiotics and zinc as one of the trace elements which is essential for many vital processes [6] and with previously demonstrated antibiofilm efficacy [8]. Consequently, the current study aimed to explore the antimicrobial interaction pattern between zinc sulfate and different classes of antibiotics including cephalosporins, fluoroquinolones, aminoglycosides, and carbapenems against biofilm-forming *K. pneumoniae*. The capabilities of such antibiotics either alone or in combination with zinc to modulate the transcriptomic profile of *K. pneumoniae* biofilm-related-genes (*mrkA, luxS, pga, wbbM,* and *wzm*) were also investigated.

## Materials and methods

### Bacterial strain and antibiotics

Previously identified biofilm-forming *K. pneumoniae* clinical isolate (BKP-122) was employed as a bacterial model in the current study [9] and it was kindly provided by Dr. Mahmoud A.F. Khalil (Department of Microbiology and Immunology, Faculty of Pharmacy, Fayoum University, Egypt). Bacterial culture was prepared from glycerol stock using nutrient-rich Luria-Bertani (LB) broth supplemented with 1% w/v glucose and incubated at 37 °C for 18 h with shaking (180 rpm). *K. pneumoniae* was streaked on tryptic soy agar (TSA) plates and further incubated for 24 h at 37 °C. Ensuring the ability of the selected isolate to produce biofilm was carried out according to Vuotto et al. using the crystal violet assay method [7]. Zinc sulfate monohydrate was purchased from Thermo-Fisher Scientific (Waltham, MA, USA). Fourteen antibiotics (ceftazidime pentahydrate, ceftriaxone sodium, cefotaxime sodium, cefepime dihydrochloride monohydrate, cefpirome sulfate, gatifloxacin, ciprofloxacin, moxifloxacin hydrochloride, ofloxacin, kanamycin sulfate, gentamicin sulfate, ertapenem sodium, meropenem trihydrate) were obtained from Sigma-Aldrich (St. Louis, MO, USA). Antibiotic stocks were prepared and diluted as per the Clinical and Laboratory Standards Institute (CLSI) requirements [10], aliquoted, and stored at – 80 °C.

### Minimum inhibitory concentrations (MICs)

The minimum inhibitory concentrations of the tested antibiotics as well as zinc sulfate were determined using the broth microdilution method [10]. The bacterial suspension was prepared and diluted using cationic adjusted Mueller-Hinton broth (CAMHB) to match the turbidity of half McFarland standard (1.5 × 10^8^ colony forming unit (CFU)/ml). In 96-well microtiter plates, antibiotics, as well as zinc sulfate, were two-fold serially diluted to reach a final concentration range of 0.125–8 mg/ml using CAMHB. Plates were inoculated with *K. pneumoniae* suspension at a final count of 5x10^5^ CFU/ml. Positive control wells were left free from the tested agents, while other wells without bacterial inoculums were considered negative controls. MIC was regarded as the minimum concentration of each of the tested agents that exhibited no bacterial growth following 18 h incubation at 37 °C using resazurin indicator, where the color change of the resazurin from purple to pink or colorless indicates bacterial growth [11].

### Biofilm susceptibility testing

#### Minimum biofilm inhibitory concentrations (MBICs)

Bacterial inoculum matching 0.5 MacFarland was diluted 100 times using LB broth and inoculated (200 µl/well) in microtiter plates. Negative control wells were inoculated with the medium only. The ability of the bacterial isolate to form biofilm following 18 h incubation was previously ensured using crystal violet assay method [7]. Plates were incubated for 18 h to allow biofilm formation followed by gentle aspiration of the medium and washing with saline twice. Plates with established biofilms were inoculated with two-fold serially diluted antibiotics at a final concentration range of 0.25–5120 µg/ml, whereas other wells were inoculated with the antibiotics in combination with 0.25 MIC of zinc sulfate (1 mg/ml). Positive control wells (inoculated with bacterial suspension only) were taken into consideration. Plates were 18 h incubated and the MBIC was recorded as the minimum concentration for each antibiotic alone and in combination with zinc sulfate that exhibited no bacterial growth [12] using resazurin as an indicator [11]. MBIC is the minimum concentration that prevented the release of planktonic cells out of the bacterial biofilm [13].

#### Effect of zinc sulfate on the transcriptomic profile of* K. pneumoniae* biofilm formation genes

The existence and the transcriptomic profile of *K. pneumoniae* biofilm formation genes (*luxS*, *mrkA*, *pgaA*, *wzm*, and *wbbM*) were determined using real-time PCR (Table 1). Bacterial inoculum was prepared using previously cultured *K. pneumoniae* to reach the mid-log phase (18 h). The bacterial suspension was further diluted in LB broth (supplemented with 1% glucose) to an OD_600_ of 0.01 and inoculated in 12-well flat-bottomed cell culture plates.

**Table 1:** Primer sequences for evaluation of the expression levels of biofilm formation genes

Gene	Primer sequence	Tm (°C)	References
*luxS*-F	5ʹ-AGT GAT GCC GGA ACG CGG-3ʹ	60	[7]
*luxS*-R	5ʹ-CGG TGT ACC AAT CAG GCT C-3ʹ	60	[7]
*mrkA*-F	5ʹ-ACG TCT CTA ACT GCC AGG C-3ʹ	60	[7]
*mrkA*-R	5ʹ-TAG CCC TGT TGT TTG CTG GT-3ʹ	60	[15]
*pgaA*-F	5ʹ-GCA GAC GCT CTC CTA TGT C-3ʹ	60	[7]
*pgaA*-R	5ʹ-GCC GAG AGC AGG GGA ATC-3ʹ	60	[7]
*wbbM*-F	5ʹ-ATG CGG GTG AGA ACA AAC CA-3ʹ	60	[7]
*wbbM*-R	5ʹ-AGC CGC TAA CGA CAT CTG AC-3ʹ	62	[7]
*wzm*-F	5ʹ-TGC CAG TTC GGC CAC TAA C-3ʹ	62	[7]
*wzm*-R	5ʹ-GAC AAC AAT AAC CGG GAT GG-3ʹ	62	[7]
*23S rRNA*-F	5ʹ-ATC GTA CCC CAA ACC GAC AC-3ʹ	62	[7]
*23S rRNA*-R	5ʹ-TTC TCC CGA AGT TAC GGC AC-3ʹ	62	[7]

*Tm* melting temperature

Plates were inoculated with 0.5 MBIC of each antibiotic in combination with ZnSO_4_ (0.25 MIC—1 mg/ml), while other wells were treated with the same concentration of the tested antibiotic only_._ In the meantime, the effect of ZnSO_4_ (0.25 MIC) was examined by adding it alone to the corresponding wells at a final concentration of 1 mg/ml, whereas positive control wells were left untreated. Plates were incubated for 18 h at 37 °C under static conditions to allow biofilm formation. The contents of all wells were gently aspirated and rinsed two times with phosphate buffer saline and the formed biofilms were detached from the plate’s surface and collected by centrifugation. A lysis solution was used to pre-treat the collected biofilms. RNA was extracted from treated cells during the biofilm development phases in addition to the positive control cells using Gene JET RNA Purification Kit (Thermo Fisher Scientific, Waltham, USA) as recommended by the producer.

Utilizing a NanoDrop spectrophotometer, the RNA yield was measured. According to the manufacturer’s guidelines, DNA contamination was eliminated using DNaseI (Thermo-Fisher Scientific, Waltham, MA, USA). The purified RNA was reverse transcripted into cDNA and amplified using TOPreal one-step RT qPCR Kit—SYBR Green with low ROX (Enzynomics, Korea). Normalization of the expressed levels of mRNA was performed using the 23S rRNA gene as an internal control gene. Real time-PCR (RT-PCR) was performed using 1 µl of diluted RNA (10 ng/ µl) for each gene, along with 10 µl of TOPreal one-step RT qPCR Reaction Mix and 1 µl of TOPreal one-step RT qPCR Enzyme Mix in addition to 1 µl of each primer (10 pmol/µl) in a final volume of 25 µl reaction. All reagents except for the diluted RNA were utilized for the negative controls.

The 2-∆∆cycle threshold (${2}^{-\Delta \Delta {\text{C}}_{\text{T}}}$) method was applied to calculate the relative gene expression as compared to untreated control using the following equations [14]. Results were presented as a fold change in the expression level of each gene and normalized to the internal control gene (*23S rRNA*).

1 $${2}^{-\Delta \Delta {\text{C}}_{\text{T}}} = [\left({C}_{\text{T}}\;\text{gene}\;\text{of}\;\text{interest}- {C}_{\text{T}}\;\text{internal}\;\text{control}\right)\text{ \,sample\, A}- [({C}_{\text{T}}\;\text{gene}\;\text{of}\;\text{interest}-{C}_{\text{T}}\;\text{ internal}\;\text{control})\;\text{sample}\;\text{B})]$$

Sample A: treated sample, Sample B: untreated sample.

2 $$\text{Fold\, change\, due\, to\, treatment }= -1 / {2}^{-\Delta \Delta {\text{C}}_{\text{T}}}$$

### Effect of different treatments on the bacterial growth

The bacterial growth rate was determined in order to evaluate the growth inhibitory potentials of the tested agents at the tested concentrations to exclude any possible effect on biofilm due to reduced bacterial growth. Briefly, an 18 h bacterial culture was adjusted to an OD_600_ of 0.01 using nutrient-rich LB broth and inoculated in 96-well flat-bottomed plates. Plates were treated with zinc (0.25 MIC) and tested antibiotics (0.5 MBIC) alone and in combinations. The bacterial growth was evaluated turbidimetrically in independent triplicates by measuring the OD600 using ELx800, Biotek (Winooski, VT, USA) at 2, 4, 6, 18, 20 and 24 h time intervals and compared to the control wells [16].

### Statistical analysis

The data acquired by the quantitative biofilm formation experiment were analyzed using the student’s t-test using GraphPad Prism software (GraphPad Software, San Diego, CA, USA). The differences were regarded as statistically significant at P < 0.05, and the confidence level was 95%.

## Results

### Antibiotic susceptibility pattern

The susceptibility profile of the tested *K. pneumoniae* isolate demonstrated antibiotic resistance to ceftazidime, ceftriaxone, cefotaxime, cefepime, kanamycin, and gentamicin in addition to intermediate sensitivity to ofloxacin. Whereas it was sensitive to gatifloxacin, ciprofloxacin, amikacin, and meropenem. Recorded data showed that among the four tested classes of antimicrobial agents, the selected *K. pneumoniae* isolate was resistant to at least one antibiotic from three different antibiotic classes (cephalosporins, aminoglycosides, and carbapenems), thus it was considered multidrug-resistant (Table 2). In the meantime, the MIC of zinc sulfate was recorded at a value of 4 mg/ml.

**Table 2:** Antibiotic and antibiofilm susceptibility patterns

Antibiotic	Class	MIC (µg/ml)	Susceptibility	MBIC (µg/ml)		Fold change	Combination efficacy
				Antibiotic	Antibiotic + ZnSO_4_		
Ceftazidime	Cephalosporins	16	R	512	32^a^	16	Synergism
Ceftriaxone	Cephalosporins	1024	R	2560	128^a^	20	Synergism
Cefotaxime	Cephalosporins	64	R	512	128^a^	4	Synergism
Cefepime	Cephalosporins	128	R	256	64^a^	4	Synergism
Cefpirome	Cephalosporins	32	-	512	64^a^	8	Synergism
Gatifloxacin	Fluoroquinolones	0.5	S	4^a^	16	4	Antagonism
Ciprofloxacin	Fluoroquinolones	0.5	S	4^a^	16	4	Antagonism
Moxifloxacin	Fluoroquinolones	4	-	8^a^	16	2	Antagonism
Ofloxacin	Fluoroquinolones	4	I	8^a^	64	2	Antagonism
Kanamycin	Aminoglycosides	128	R	512	128^a^	4	Synergism
Gentamicin	Aminoglycosides	16	R	64	32^a^	2	Synergism
Amikacin	Aminoglycosides	4	S	8	2^a^	4	Synergism
Ertapenem	Carbapenems	4	R	8	1^a^	8	Synergism
Meropenem	Carbapenems	0.0625	S	0.5^a^	2	4	Antagonism

The susceptibility profile was based on CLSI [17]

*R* resistant, *I* intermediate, and *S* sensitive

^a^One-fold reductions below these concentrations were considered as sub-MBIC and applied to evaluate the transcriptomic profile of biofilm formation genes

### Antibiofilm susceptibility profile of sole different antibiotics and in combination with zinc sulfate

Regarding the antibiofilm potentials of ceftazidime, ceftriaxone, cefotaxime, cefepime, and cefpirome, the recorded results indicated that there is a gap between the MBICs compared to the corresponding MICs on planktonic cells, where an increase in the MBICs was recorded in the order of 32-, 2.5-, 8-, 2-, and 16-folds, respectively. A similar pattern was detected in the case of gatifloxacin, ciprofloxacin, moxifloxacin, and ofloxacin where they showed higher MBICs by 8-, 8-, 2-, and 2-folds, respectively. Also, kanamycin, gentamicin, amikacin, ertapenem, and meropenem exhibited elevated MBICs by 4-, 4-, 2-, 2-, and 8-folds, respectively.

Results revealed that zinc sulfate at a concentration of 0.25 MIC (1 mg/ml) potentiates the antibiofilm effectiveness of ceftazidime, ceftriaxone, cefotaxime, cefepime, and cefpirome, where the MBIC decreased by a value of 16-, 8-, 4-, 4- and 8-folds, respectively. In the meantime, an enhancement in the antibiofilm potentials of kanamycin, gentamicin, amikacin, and ertapenem was recorded following combination with zinc sulfate in the order of 4-, 2-, 4- and 8-folds, respectively. On the other side, a reduction in the antibiofilm efficacies was observed following the combination between zinc sulfate and gatifloxacin, ciprofloxacin, moxifloxacin, ofloxacin, and meropenem by 4-, 4-, 2-, 2- and 4-folds, respectively. One dilution below the recorded MBIC of each of the tested antibiotics either in the presence or in the absence of zinc sulfate, where it confirms the biofilm formation (sub-MBIC), was further applied to examine the transcriptomic profile of biofilm formation genes as indicated in Table 2.

### Effect of zinc sulfate on the transcriptomic profile of *K. pneumoniae* biofilm formation genes

**Fig. 1:**
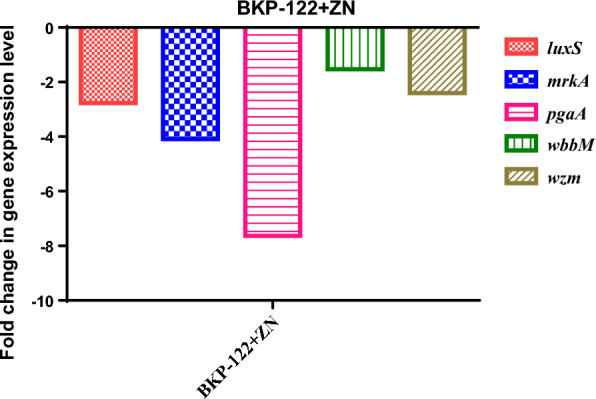
Gene expression profile of *K. pneumoniae* biofilm formation genes presented as fold change in the expression level post-exposure to zinc sulfate as compared to the control bacterial cells. *BKP-122* biofilm forming *K. pneumoniae* clinical isolate, *ZN* zinc sulfate

In comparison to the control cells, the impact of various antibiotic concentrations (sub-MBIC) alone and in combination with zinc sulfate (0.25 MIC—1 mg/ml) against *K. pneumoniae* biofilm was assessed by comparing the expression profile of genes controlling biofilm formation using RT-PCR. Current findings demonstrated that zinc sulfate alone reduced the expression of biofilm-correlated genes *luxS*, *mrkA*, *pgaA*, *wbbM,* and *wzm* genes by 2.8-, 4.1-, 7.6-, 1.5- and 2.4-folds, respectively as compared to the control bacterial cells (Fig. 1).

**Fig. 2:**
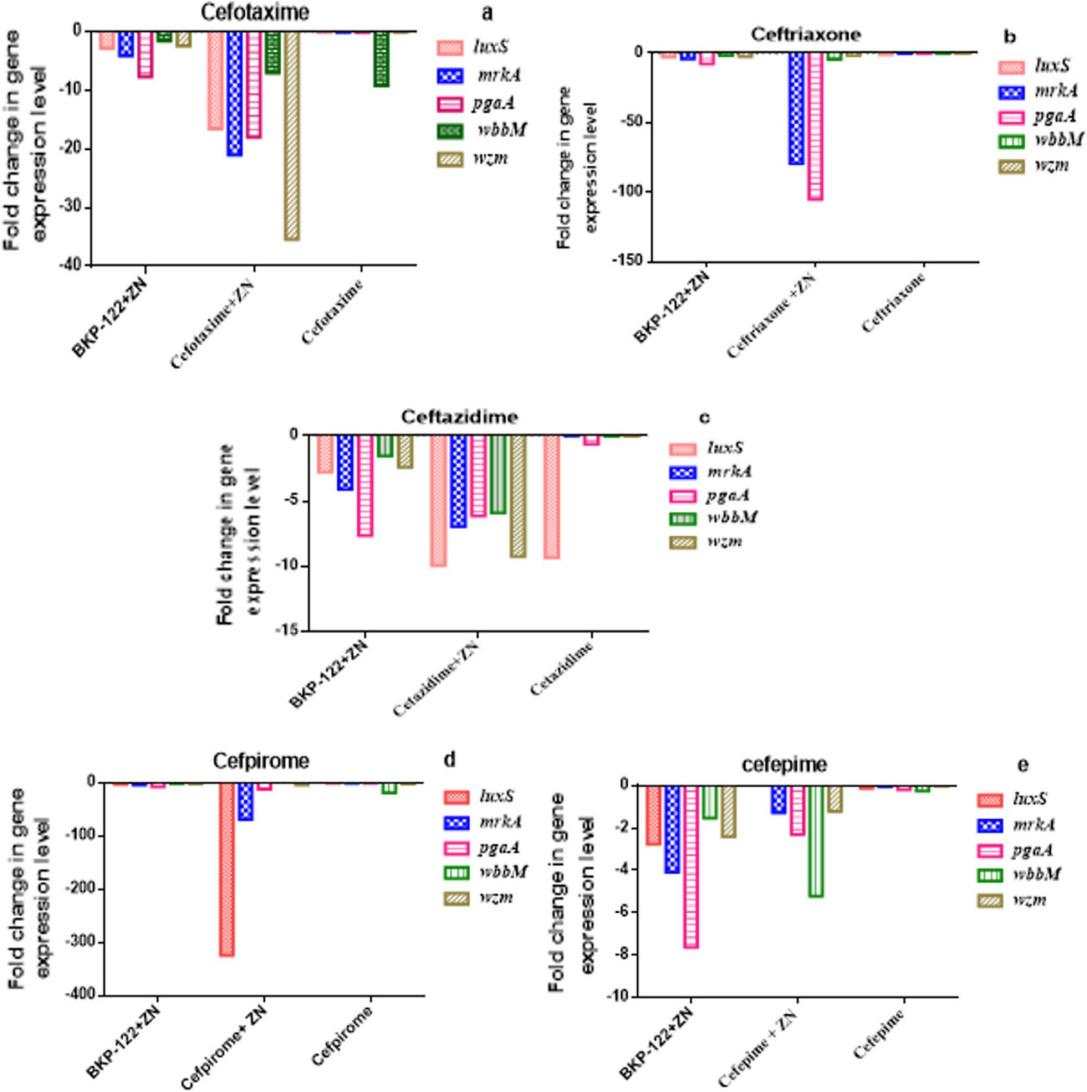
Transcriptomic profile of *K. pneumoniae* biofilm-associated genes expressed as fold change in the gene expression level post-treatment with **a**: cefotaxime, **b**: ceftriaxone, **c**: ceftazidime, **d**: cefpirome, and **e**: cefepime alone and in combination with zinc sulfate

Similarly, the examined biofilm-related genes were down-regulated following treatment with different classes of antibiotics. In this context, zinc sulfate enhanced the anti-biofilm efficacies of cephalosporins (e.g. cefotaxime, ceftriaxone, ceftazidime, cefpirome, and cefepime) via down-regulating the expression of biofilm-related genes in the order of 18-, 38-, 5-, 77- and 2-folds, respectively. Concerning the effect of zinc sulfate/cefotaxime combination on *K. pneumoniae* biofilm-associated genes, it was found that the expression levels of *luxS*, *mrkA*, *pgaA*, *wbbM,* and *wzm* genes were down-regulated by 16.5-, 21-, 18-, 7- and 35.4-folds as compared to that observed following exposure to cefotaxime alone, respectively. Also, zinc sulfate/ceftriaxone combination reduced the expression of *luxS*, *mrkA*, *pgaA*, *wbbM,* and *wzm* genes by 2.7-, 79.3-, 105-, 4.3-, and 1.7-folds, respectively. Moreover, the expression levels of the tested genes were decreased in the order of 9.9-, 6.9-, 6.1-, 5.9- and 9.3-folds following treatment with zinc sulfate and ceftazidime in combination, respectively. Analogously, zinc sulfate/cefpirome combination down-regulated the expression of *luxS*, *mrkA*, *pgaA*, *wbbM,* and *wzm* genes by 324.7-, 68.8-, 11.9-, 1.7- and 3.7-folds, respectively. Furthermore, the expression levels of *luxS* and *wbbM* genes were reduced by values of 4.3-, and 5.2-folds post-exposure to zinc sulfate/cefepime combination compared to that observed following treatment with cefepime alone, respectively (Fig. 2).

**Fig. 3:**
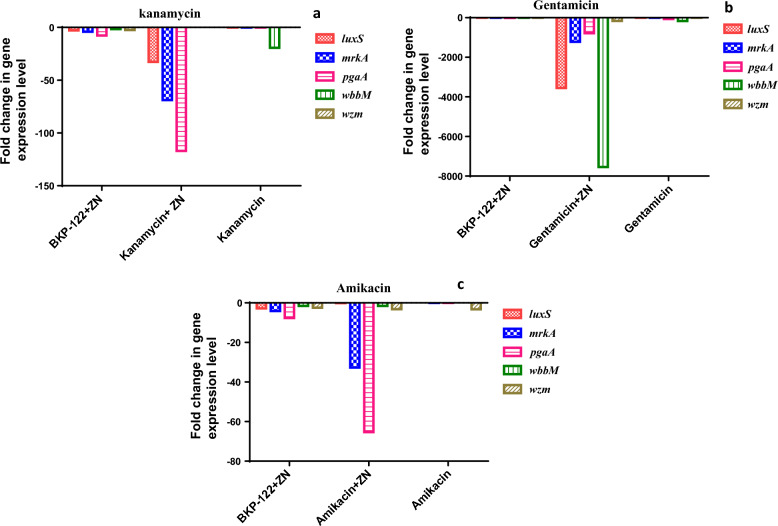
Expression pattern of *K. pneumoniae* biofilm-correlated genes presented as fold change following exposure to zinc sulfate in combination with **a**: kanamycin, **b**: gentamicin, and **c**: amikacin compared to sole treatment with antibiotics

Regarding the transcriptomic modulatory potentials of zinc sulfate in combination with aminoglycosides (e.g. kanamycin, gentamicin, and amikacin), results revealed that zinc sulfate in combination with the tested aminoglycosides could reduce the expression of biofilm-elated genes by 40-, 2602- and 20-folds, respectively. The recorded data showed that zinc sulfate/kanamycin combination down-regulated the expression levels of *luxS*, *mrkA*, *pgaA*, *wbbM,* and *wzm* genes by 32.7-, 68.8-, 116.9-, 1.7- and 3.7-folds, respectively as compared to that with kanamycin alone. Moreover, zinc sulfate/gentamicin combination reduced the expression of *luxS*, *mrkA*, *pgaA*, *wbbM,* and *wzm* genes in the order of 3548.3-, 1214.2-, 785.5-, 7541.3- and 163.1-folds, respectively as compared to that with gentamicin alone. Additionally, zinc sulfate/amikacin combination exhibited down-regulation of the expression levels of *luxS*, *mrkA*, *pgaA*, *wbbM,* and *wzm* genes by values of 0.12-, 32.7-, 65.3-, 1.5- and 3.2-folds as compared to that with amikacin alone, respectively (Fig. 3).

**Fig. 4:**
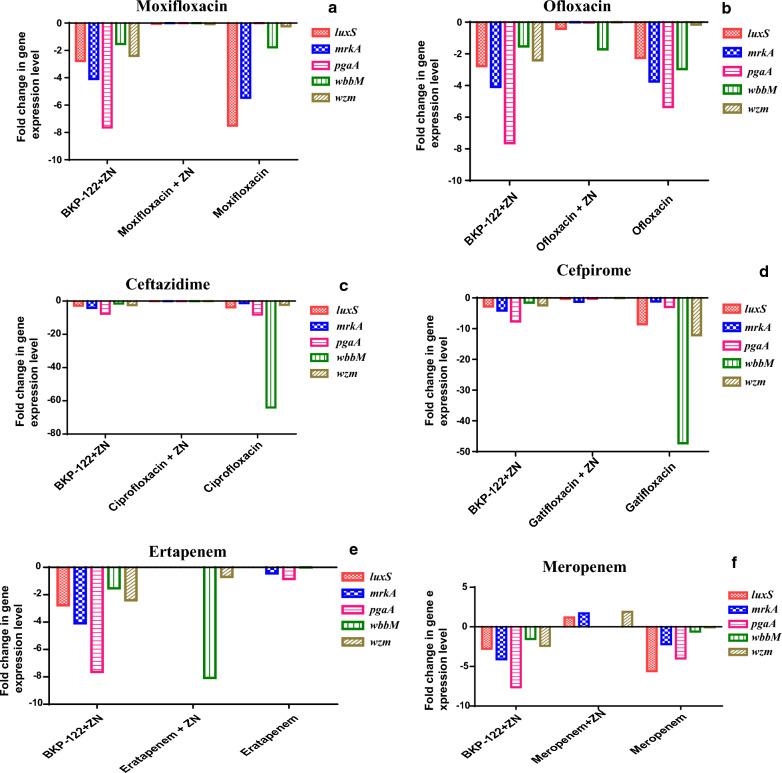
Gene expression levels of *K. pneumoniae* biofilm-associated genes following treatment with sole **a**: moxifloxacin, **b**: ofloxacin, **c**: ciprofloxacin, **d**: gatifloxacin), **e**: ertapenem, and **f**: meropenem as well as in combination with zinc sulfate

On the other side, the tested fluoroquinolones (moxifloxacin, ofloxacin, ciprofloxacin, and gatifloxacin) in combination with zinc sulfate showed an antagonistic effect. That was apparent in that the tested antibiotics alone showed down-regulatory potentials on the biofilm-related genes, however, the combination of these antibiotics with zinc sulfate exhibited a reduction in this down-regulation by 2-, 2-, 15- and 14-folds, respectively. Recorded results demonstrated that zinc sulfate in combination with moxifloxacin antagonizes the antibiofilm potentials of moxifloxacin via reducing the down-regulation of the expression levels of *luxS*, *mrkA*, *pgaA*, *wbbM,* and *wzm* genes by 0.057–0.0025-, 0.00016-, 0.0038- and 0.08-folds compared to that observed in case of moxifloxacin, respectively. A comparable pattern was detected in the case of zinc sulfate/ofloxacin combination, where the expression levels of the biofilm-related genes were reduced in the order of -0.42-, 0.00009-, 0.01-, 1.71- and -0.0035-folds compared to ofloxacin, respectively. Moreover, the down-regulation of the tested genes was decreased following the combination between zinc sulfate and ciprofloxacin by values of 0.053-, 0.0086-, 0.00033-, 0.00044- and 0.0072-folds, respectively, compared to that recorded post ciprofloxacin treatment. In the same context, zinc sulfate antagonizes the efficacy of gatifloxacin by reducing the expression of *luxS*, *mrkA*, *pgaA*, *wbbM,* and *wzm* genes by 0.26-, 1.247-, 0.223-, 0.000001- and 0.0068-folds as compared to that observed in case of gatifloxacin alone, respectively (Fig. 4).

Concerning the effect of zinc sulfate on carbapenems, the results revealed an enhancement in the efficacy of ertapenem via down-regulating the expression levels of *luxS*, *mrkA*, *pgaA*, *wbbM,* and *wzm* genes by 5.1-, 1.7-, 3.2-, 8.1- and 0.7-folds, respectively. On the other hand, it antagonizes the effectiveness of meropenem by up-regulating the expression of *luxS*, *mrkA*, *pgaA*, *wbbM,* and *wzm* genes by 1.2- 1.7-, 0.0003-, 0.009- and 1.9-folds, respectively (Fig. 4).

### Effect of different treatments on the bacterial growth

**Fig. 5:**
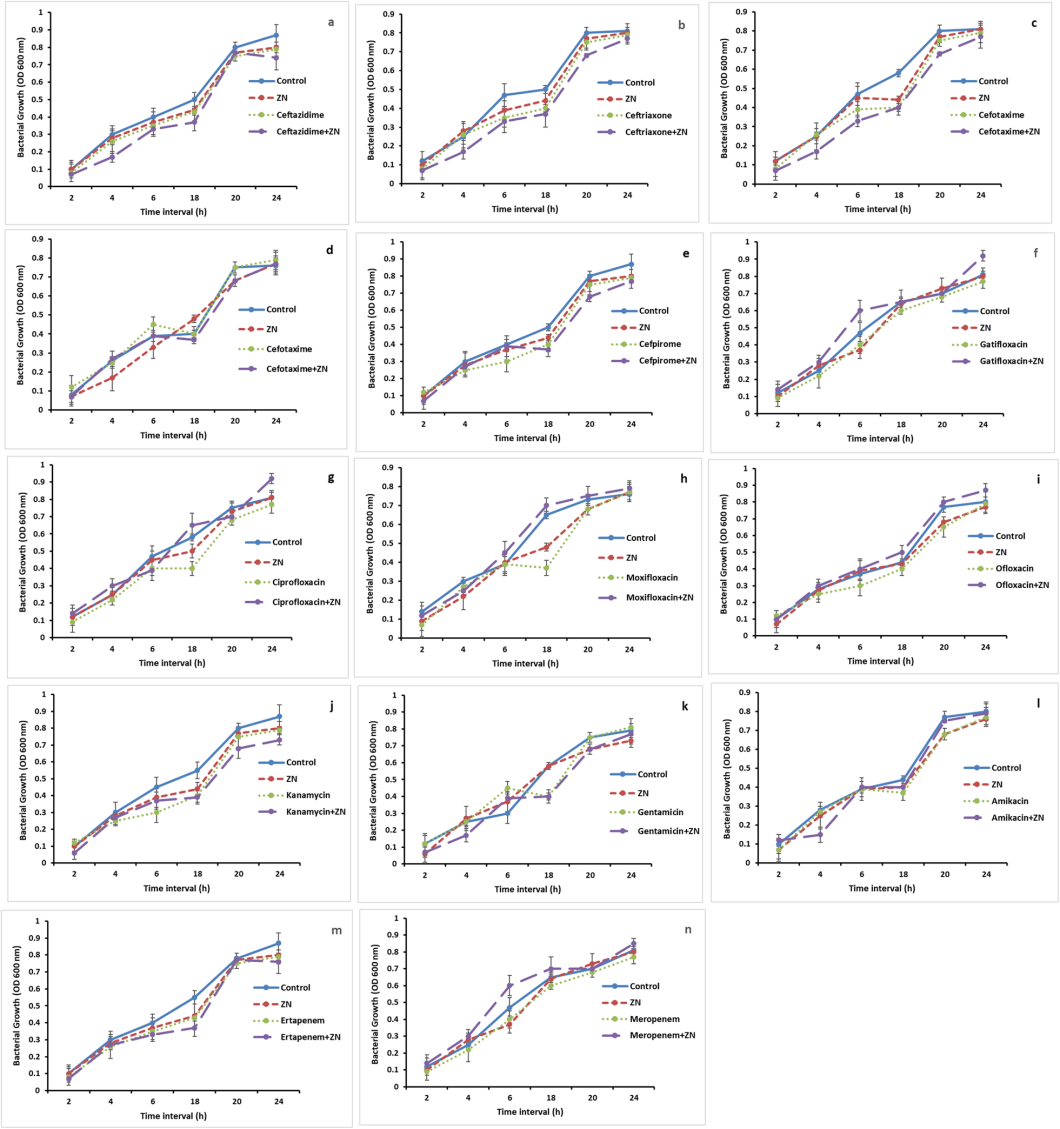
Bacterial growth curve of *K. pneumoniae* isolate following treatment with zinc sulphate and different antibiotics as well as zinc-antibiotic combinations at different time intervals compared to the control

Recorded data revealed that a non-significant difference in the growth pattern was detected at different time intervals following different treatments as compared to the control cells. Thus, the tested concentration of zinc sulphate as well as antibiotics either alone or in combination didn’t show considerable inhibitory effect on the growth of the tested isolate during the formation of the biofilm as compared to the control cells (Fig. 5).

## Discussion

*K. pneumoniae* is regarded as one of the most crucial pathogens causing nosocomial infections in healthcare settings. It is the major causative agent of infections in critical sites such as the respiratory and urinary tract in addition to the biliary duct as well as the surgical wounds [7]. Antibiotics are the major treatment applied for these types of infections and in the meantime, zinc supplements are commonly prescribed in clinical settings due to the known ability of zinc to enhance the adaptive immune response (antibody-mediated) against different pathogens [18]. Consequently, the current study aimed to investigate the antimicrobial interaction pattern between zinc sulfate in combination with different classes of antibiotics against *K. pneumoniae* biofilm formation on the phenotypic and genotypic levels. Therefore, the MICs of different antibiotics as well as zinc sulfate were determined. The antibiotic susceptibility pattern revealed that the selected *K. pneumoniae* clinical isolate (BKP-122) is MDR. MDR was based on resistance to at least one antibiotic from three different antibiotic classes, namely cephalosporins, aminoglycosides, and carbapenems. Additionally, 0.25 MIC of zinc sulfate (1 mg/ml) was applied in the present study, where this concentration of zinc sulfate has a negligible effect on planktonic cells and consequently its antibiofilm potential on the tested bacterial isolate would be null. A comparable concentration of zinc sulfate was also applied in another study to investigate the effect of zinc in combination with different antibiotics against *Pseudomonas aeruginosa* biofilms [6].

Biofilm eradication is challenging, where the existence of the metabolically inactive persister cells in the matrix of the bacterial biofilm renders these bacteria unable to be destroyed by antimicrobials [19]. This justifies the recorded gap between the MICs and the MBICs of the tested antimicrobial agents and reflected the importance of testing their antibiofilm potentials. It is well-known that the bacterial cells in biofilms are more resistant to antibiotics and hence more prone to treatment failure compared to planktonic cultures. This notion is of great clinical importance since the clinically required concentrations to eradicate biofilms are far from the therapeutically achievable ones [20]. A recent study demonstrated an association between antibiotic resistance and biofilm formation ability in *K. pneumoniae,* where the isolates capable of biofilm formation were found to be MDR compared to non-biofilm producers. That was probably correlated to that the bacterial plasmids carrying antibiotic-resistance genes could also express genes participating in bacterial adherence as well as biofilm formation [21].

Despite that, the antibiofilm effectiveness of zinc has not been fully recognized but zinc may exert selective pressure on bacteria that can produce biofilm via different mechanisms. Zinc could react with the constituents of the bacterial biofilm such as poly-n-acetylglucosamine (PGA). On the other side, the ability of zinc to inhibit the bacterial biofilms of other microorganisms that lack PGA in their biofilms indicated that it is not the only inhibitory mechanism [8]. Another study suggested that the chelation between zinc and extracellular DNA as a component of the biofilm matrix could affect biofilm stability [22]. It was also demonstrated that zinc could impair *E. coli* and *K. pneumoniae* biofilms by hindering iron uptake which is essential in biofilm formation [23]. Zinc was reported to interfere with the toxin-antitoxin system which contributed to biofilm formation and prevented the expression of adhesion factors in addition to the exopolysaccharides of the bacterial biofilms [24]. In addition to the previously reported mechanisms concerning the antibiofilm potentials of zinc, our study proved the effectiveness of zinc alone against *K. pneumoniae* biofilm via the down-regulation of biofilm-related genes. In the mean context, the tested cephalosporins as well as aminoglycosides in addition to ertapenem in combination with zinc down-regulated the expression levels of biofilm-associated genes.

Regarding the ability of zinc to enhance the antibacterial effectiveness of ceftazidime as an example of the third-generation cephalosporins, it was demonstrated that three active groups were responsible for the antibacterial activity of ceftazidime. It was reported that the increased affinity to penicillin-binding proteins of Gram-negative bacteria is brought on by the aminothiazole ring as well as the propyl-carboxy group at position 7 of ceftazidime. The pyridine group in position 3 of the ceftazidime molecule, which promotes intra-bacterial penetration in a short time, is another active moiety. It was found that the formation of a stable Zn-antibiotic complex by the chelation of Zn with the propyl-carboxy group (at position 7) of ceftazidime could facilitate its entrance into the bacterial cells and consequently enhance its antimicrobial potential [25].

In this study, the reduced antibiofilm activity of fluoroquinolones in combination with zinc but not in the case of aminoglycosides could be attributed to the ability of fluoroquinolones to chelate zinc ions. A study reported that zinc combination with ciprofloxacin reduced the effectiveness of ciprofloxacin against *E. coli* due to the chelation interaction between zinc and ciprofloxacin*.* On the other hand, gentamicin as a non-fluoroquinolone antibiotic that cannot chelate zinc ions did not demonstrate a comparable effect in presence of zinc ions due to the lack of zinc-gentamicin interaction [26]. It was proposed that this could be attributed to the reduced ability of fluoroquinolones to penetrate into the bacterial cells which is a function of the antibiotic net charge. This net charge was found to be greatly affected following complex formation with divalent ions, particularly the chelation between metal ion and the 4-oxo adjacent carboxyl groups, thus hindering the bacterial uptake of fluoroquinolones [27].

On the molecular level, current findings demonstrated consistency with the results on the phenotypic level where zinc in combination with fluoroquinolones down-regulated *K. pneumoniae* biofilm-related genes by a reduced level as compared to that in the case of fluoroquinolones alone. It was reported that the coexistence of two stress factors as antibiotics in addition to metal ions could play an alarming role in the de novo development of resistance to antibiotics, especially in the complicated bacterial populations where there is an elevated chance for increased rates of mutation and the transfer of resistance genes horizontally [28]. Furthermore, the rate of transmission of these resistance genes might be affected by the existence of metal ions [29].

The CLSI reported that Enterobacteriaceae which produce carbapenemase are usually showing intermediate susceptibility or resistance to one or more carbapenem as well as resistance to one or more of the third-generation cephalosporins considering that the resistance to ertapenem highly indicates the production of carbapenemase [17]. Thus, carbapenemase production might contribute to the ertapenem resistance in the present study.

*K. pneumoniae* is characterized by its ability to produce metallo-b-lactamases (MBLs) which play a key role in the resistance to carbapenems although other mechanisms are involved in this resistance. MBLs are transmissible carbapenemases that depend on zinc ions in their activity. A recent study reported that zinc chelators could restore the antibacterial activity of meropenem against *K. pneumoniae* by acting as an inhibitor to MBLs [30]. That could be of great importance in justifying the recorded antagonism that was observed in the current study when zinc was combined with meropenem, where the presence of zinc could act as an inducer for the production of MBLs leading to an antagonistic effect of meropenem in combination with zinc.

On the other side, the detected synergism in the case of ertapenem in combination with zinc could be related to the demonstrated variability in the affinity of zinc to different MBLs [31], taking into consideration that the tested *K. pneumoniae* strain showed sensitivity to meropenem and on the contrary, it was resistant to ertapenem. Such finding could indicate that ertapenem might be inactivated by a resistance mechanism which was not applicable in the case of meropenem as there was no resistance against it in the current study particularly since our results on the molecular level were coherent with that on the phenotypic level. In the meantime, evaluating the bacterial viability following different treatments indicated that the recorded difference in gene expression is not due to the effect of different treatments on the bacterial growth rate.

To the best of our knowledge, this is the first report demonstrating the modulatory potentials of zinc sulfate in combination with different antibiotic classes on the transcriptomic profile of MDR *K. pneumoniae* biofilm formation genes. The current findings are of critical interest, especially in the case of the reported antagonism between some antibiotics in combination with zinc against *K. pneumoniae* biofilm. This issue will be more problematic in patients who receive zinc as a supplement [32] as it could worsen the patients’ syndrome rather than overcoming bacterial resistance.

## Conclusions

The present study revealed a variable range of the modulatory potentials of zinc sulfate in combination with different antibiotics against MDR *K. pneumoniae* biofilm on both the phenotypic and genotypic levels with a potential enhancement of the antibiofilm effectiveness in the case of cephalosporins, aminoglycosides as well as ertapenem. On the other hand, a significant antagonism was detected following the combination of zinc sulfate with fluoroquinolones and meropenem.

## Data Availability

All data generated or analyzed during this study are included in this published article and its additional information file.

## Abbreviations

*K.** pneumoniae*: *Klebsiella pneumoniae*
BKP: Biofilm-forming *K. pneumoniae*
MDR: Multidrug-resistant
LB: Luria–Bertani
TSA: Tryptic Soy Agar
CAMHB: Cationic adjusted Mueller Hinton broth
CFU: Colony forming unit
CLSI: Clinical and Laboratory Standard Institute
MIC: Minimum inhibitory concentration
MBIC: Minimum biofilm inhibitory concentration
Real time-PCR: Real time-PCR
PGA: Poly-N-acetylglucosamine
MBLs: Metallo-b-lactamases
